# Mother-Child Neurophysiological Synchrony Moderates the Relation Between Maternal Affect Experiences and Child Emotion Dysregulation

**DOI:** 10.1007/s10802-025-01380-4

**Published:** 2025-10-20

**Authors:** Molly E. Hale, Kayley E. Morrow, Grace Steffen, Haobi Wang, Christian M. Jerry, Jianjie Xu, Zhou Rachel Han, Drew Abney, Cynthia Suveg

**Affiliations:** 1 Department of Psychology, University of Georgia, Athens, GA USA; 2 Beijing Key Laboratory of Applied Experimental Psychology, National Demonstration Center for Experimental Psychology Education, National Virtual Simulation Center for Experimental Psychology Education, Faculty of Psychology, Beijing Normal University, Beijing, China

**Keywords:** Mother-child, Affect, Emotion dysregulation, RSA, DlPFC, Synchrony

## Abstract

The parent-child emotion regulation dynamics model posits that child emotion regulation develops in the context of the parent-child relationship across multiple levels of functioning. Such a conceptualization dovetails newer conceptualizations of psychopathology, which argue that to most effectively understand and treat psychological problems, evaluation of individual and dyadic indicators across behavioral, physiological, and neural indicators is crucial. The present study adopted this multimethod, multilevel approach to examine the interacting role of maternal affect experiences with dyadic neurophysiological processes in relation to child emotion dysregulation – a transdiagnostic risk factor for both child internalizing and externalizing psychopathology. Eighty mothers (*M*_*age*_ = 35.97, *SD* = 5.46) and their school-age children (*M*_*age*_ = 5.88, *SD* = 0.80; 54% girl; 46% boys) completed a dyadic stress task. Across the entire task, respiratory sinus arrythmia (RSA) and dorsolateral prefrontal cortex (dlPFC) activation were measured for both mothers and children. Mother-child temporal concordance (i.e., synchrony) was then computed for each modality. Mothers also reported on their own affect experiences and children’s emotion dysregulation. In the context of positive RSA synchrony and positive left and right dlPFC synchrony, maternal positive affect experience was most strongly and negatively associated with child emotion dysregulation. Conversely, the relation between maternal positive affect and child emotion dysregulation was negative and weakest in the context of negative RSA synchrony and left dlPFC synchrony. Findings provide empirical support for the parent-child emotion regulation dynamics model, suggesting that multilevel synchrony processes have significant implications for child emotion dysregulation, a robust transdiagnostic indicator of psychopathology.

**Supplementary Information:**

The online version contains supplementary material available at 10.1007/s10802-025-01380-4.

Emotion regulation involves the automatic and intentional processes associated with expressing and modulating emotional experiences. Emotion regulation develops largely in the context of the parent-child relationship and is critical for healthy adjustment (Eisenberg et al., 2003). On the flipside, emotion dysregulation reflects difficulties in the processes associated with the effective modulation of emotional experiences and is considered a transdiagnostic indicator of psychopathology symptoms (Beauchaine & Cicchetti, 2019). Research to date has typically focused on intra- and inter-personal *behavioral* indicators and, to a lesser extent, physiological and neural processes associated with children’s developing emotion regulation. The parent-child emotion regulation dynamics model posits that child emotion regulation is developed through a combination of parental and dyadic indices across multiple levels of functioning (e.g., physiological, neural). Thus, research must move beyond behavioral indicators and include measures of dyadic physiology and neural activation when measuring emotional functioning in children (Morris et al., 2018; Ratliff et al., 2022). Guided by this framework, the present study conducted a multimethod, multilevel examination of intra- and inter-personal processes related to child emotion dysregulation specifically. Analyzing the complex innerworkings of individual and dyadic processes in relation to child emotion dysregulation is critical given the robust correlation between emotion dysregulation and subsequent child psychological adjustment (Aldao, et al., 2016; Cuthbert & Insel, 2013; Milone & Sesso, 2024; Ratliff et al., 2022).

The parent-child emotion regulation dynamics model stems from the early work of Bronfenbrenner (1979) who proposed that child development occurs through the interaction of individual and broader contextual influences. The recommendations outlined in the extension of the parent-child emotion regulation dynamics framework emphasize exploring individual and dyadic functioning using a multilevel approach and echo the sentiments of newer conceptualizations of psychopathology (e.g., Research Domain Criteria; Cuthbert & Insel, 2013; Insel et al., 2010). More specifically, newer conceptualizations of psychopathology argue that to best identify transdiagnostic indicators of psychological disorders, researchers must consider individual and dyadic functioning across domains of behavior and biology. The current study focuses specifically on emotion dysregulation (i.e., difficulties with managing one’s emotional responses) due to the construct’s inherent relation with child psychopathology (Beauchaine & Hinshaw, 2020). Grounded in a multilevel approach to identify a transdiagnostic indicator of psychopathology, the present study evaluated the influence of maternal affect experiences alongside dyadic physiological and neural indicators on child emotion dysregulation during the early school-age period.

Beginning with behavioral indicators, a plethora of research has linked the intrapersonal experience of maternal affect with child emotion regulation (Are & Shaffer, 2016; Morris et al., 2018; Ratliff et al., 2022; Tan et al., 2019). In fact, maternal affect experiences, both positive and negative, inform how mothers interact with their children, which ultimately links to children’s emotion regulation (Kerns et al., 2017; Martin et al., 2002). For instance, a preponderance of maternal negative affect experiences may contribute to a cold, harsh parenting environment that undermines healthy child emotion regulation development (Eisenberg, 2020; Oshri et al., 2023; Ratliff et al., 2022). On the flipside, mothers who experience predominantly positive affect experiences often have children who exhibit decreased emotion dysregulation (Senehi & Brophy-Herb, 2020; Thomassin et al., 2017). Mothers’ positive affect experiences likely set the stage for emotional intimacy and support in parent-child interactions that in turn, scaffold adaptive emotion regulation by providing a safe space for children to express and learn emotion regulation strategies. The early school-age period is a salient time to assess influences on emotion regulation given that difficulties in this developmental domain foreshadow the subsequent onset of psychopathology (Cavicchioli et al., 2023).

## Parent-Child Neurophysiological Synchrony

Beyond maternal affect experiences, methodological advancements have allowed researchers to assess dynamic, bidirectional exchanges at the physiological and neural levels, in line with the present study’s guiding framework (Beauchaine & Hinshaw, 2020; Chan et al., 2022; Ratliff et al., 2022; Somers et al., 2024). Synchrony is a metric that can quantify the degree of concordance between parent-child neurophysiological processes (Alonso et al., 2024; Atzil & Gendron, 2017; DePasquale, 2020). Neurophysiological synchrony can be measured in terms of direction (positive versus negative) and strength (strong versus weak). Positive synchrony refers to parent-child indicators of neurophysiological activity that move in the same direction over time, whereas negative synchrony suggests that indicators move together in opposite directions over the course of a task (Feldman, 2021). Strong synchrony means parent and child indices are closely related, moving together across time either in a positive or negative direction, whereas weak synchrony implies a limited degree of concordance between individual activation indicators. In this way, positive synchrony may exacerbate risk associated with the experience of mother negative affect whereas negative or no synchrony may act as a buffer (Davis et al., 2018). Despite these proposed relations, such associations have only been examined within a behavioral context, leaving questions regarding how synchrony may influence maternal affect experiences and child emotion dysregulation using neurophysiological indices.

### Physiological Synchrony

Respiratory sinus arrhythmia (RSA) is conceptualized as a measure of respiratory modulation of vagal control of the heart (i.e., cardiac vagal tone; Grossman & Taylor, 2007; Perry et al., 2018). RSA measures the intersection between the cardiovascular and respiratory systems and is considered a proximal measure of one’s parasympathetic nervous system functioning with implications for individual self-regulation (Grossman & Taylor, 2007). RSA synchrony is an indicator of the degree of parent-child mutual responsiveness to a stressful and/or social interaction task (Davis et al., 2018). However, unlike positive behavioral synchrony (e.g., shared parent-child positive affect), which is uniformly associated with positive child outcomes, relations between RSA synchrony and child outcomes are context-dependent (Hale et al., 2023; Lunkenheimer et al., 2018; Suveg et al., 2019). For example, in the context of positive synchrony, firm parental control related to increased youth externalizing problems in a sample of parent-adolescent dyads. Conversely, the relation between positive parenting and decreased behavioral problems and the association between negative parenting and increased behavioral problems were strengthened in the context of parent-child synchrony (Oshri et al., 2023). In a study of early adolescents and their mothers, maternal depressive symptoms related to elevations in youth psychopathology symptoms in the context of positive RSA synchrony. However, when RSA synchrony was negative, maternal depressive symptoms were associated with fewer symptoms of psychopathology in youth (West et al., 2020). Further, albeit not moderation analyses, one study reported low levels of maternal depressive and child internalizing symptoms related to positive RSA synchrony whereas in the context of high symptoms, mother-child RSA synchrony was observed to be negative (Suveg et al., 2019). Additionally, albeit not moderation directly, studies have reported links between RSA synchrony with broader contextual factors (i.e., income, child gender). Specifically, in a recent meta-analysis, authors reported significantly weaker RSA synchrony when parent-child dyads reported lower income (Miller et al., 2023). Further, a myriad of studies have controlled for child gender due to theorized differences in RSA synchrony by child gender (Feldman, 2021; Lunkenheimer et al., 2021a, b; Oshri et al., 2021). The present study expands empirical work by examining the moderating role of RSA synchrony on the relation between two transdiagnostic indicators of psychopathology (i.e., maternal affect experiences and child emotion dysregulation), while controlling for income and child gender.

### Neural Synchrony

Similar to RSA, activation in the dorsolateral prefrontal cortex (dlPFC) is critical for regulating emotions as well as carrying out cognitive functions and engaging in adaptive behavioral exchanges (Lee & Williams, 2024). Activation in the dlPFC has been associated with increased self-monitoring and adaptive emotional and behavioral regulation across development (Nguyen et al., 2021a, b). Research also reports differences in dlPFC activation with socioemotional functioning depending on the hemisphere: activation in the left dlPFC is associated with enhanced positive mood and decreased negative mood (Maeoka et al., 2012; Pena-Gomez et al., 2011) whereas the right dlPFC has been related to emotional processing (Sanchez-Lopez et al., 2018).

Given the implications of dlPFC activation for adaptive emotion regulation and healthy social interactions, work has begun to investigate parent-child dlPFC synchrony (Alonso et al., 2024; Nguyen et al., 2020; Ratliff et al., 2022; Zhang et al., 2017). Miller and colleagues (2019) found then when comparing dlPFC synchrony between tasks, more positive dlPFC synchrony was found between mothers and their 8- to 13-year-old children during cooperative tasks when compared to independent tasks. The same pattern of effects was found in a sample of parents and their 5- and 6-year-old children (Nguyen et al., 2020, 2024). Research has also examined direct associations between neural synchrony and child adjustment, noting significant, positive relations between positive mother-child neural synchrony with adaptive child emotion regulation, shared positive affect expression, and parental warmth across cooperative, competitive, and individual tasks (Liu et al., 2024; Miller et al., 2019; Nguyen et al., 2020; Reindl et al., 2018).

The vast majority of dlPFC synchrony work has centered around direct associations between synchrony and child adjustment. For instance, fathers with more positive perceptions of their parenting role displayed stronger dlPFC synchrony during a cooperation task with their children when compared to fathers with less positive perceptions (Nguyen et al., 2021b). Conversely, mother-preschool dyads experiencing higher levels of adversity, characterized by low income and high familial risk (e.g., household chaos), demonstrated weaker PFC synchrony during a stress task compared to dyads with lower levels of adversity (Hoyniak et al., 2021). Though work has primarily investigated direct links between neural synchrony and parent and child adjustment indicators, biobehavioral frameworks of child development suggest that parent-child synchrony processes can exacerbate or buffer risk (Ratliff et al., 2022). Further, in recent reviews of neural synchrony, Alonso and colleagues (2024) as well as Nguyen et al. (2024) argue that a gap in current research is the examination of neural synchrony as a moderator between contextual factors (e.g., maternal behaviors) and child psychological outcomes. Thus, the present study builds on foundational neural synchrony work and assesses neural synchrony as a moderator of the association between mother’s affect experiences and child emotion dysregulation.

## The Present Study

Guided by the extension of the parent-child emotion regulation dynamics model (Ratliff et al., 2022), the present study analyzed maternal affect experiences and mother-child physiological- and neural-synchrony in relation to child emotion dysregulation - a transdiagnostic indicator of psychopathology. Relations between maternal positive and negative affect experiences and child emotion dysregulation as moderated by RSA and dlPFC synchrony were assessed. Understanding the complex relations among intra- and inter-personal processes associated with children’s emotion dysregulation can have meaningful conceptual and clinical implications given the transdiagnostic nature of this construct in the prediction of youth’s psychological maladjustment. Additionally, prior synchrony work has found inconsistent findings with regard to gender and income. Some work suggests differences between boys and girls in relation to neurophysiological synchrony with their parents whereas other work does not report differences. In fact, one study notes differences in synchrony by income (Miller et al., 2023); however, with only one empirical examination, there is not enough research to draw firm conclusions. Thus, gender and income were controlled in analyses to better isolate the hypothesized effects (DePasquale, 2020; Somers et al., 2024; Xu et al., 2024b).

It was hypothesized that there would be a negative relation between maternal positive affect experiences and child emotion dysregulation which would be strongest in the context of positive RSA synchrony and weakest in the context of negative RSA synchrony. Given the lack of prior work on how dlPFC synchrony may alter influence the association between positive maternal affect experiences and child emotion dysregulation, no specific hypotheses were put forth for this specific moderation. It was hypothesized that a positive relation between maternal negative affect experiences and child emotion dysregulation would be strongest in the context of positive RSA and dlPFC synchrony and weakest in the context of negative RSA and dlPFC synchrony. When considering the lateralization of the dlPFC, it was hypothesized that relations would be stronger for left dlPFC when compared to right dlPFC synchrony given data suggesting ties between activation in this brain region and affect (Lee & Williams, 2024; Monsell, 2003).

## Method

### Participants

Participants included 80 mothers (*M*_*age*_ = 35.97, *SD* = 5.46) and children (*M*_*age*_ = 5.88, *SD* = 0.80; 54% girl; 46% boys). Children were required to be between the ages of 5- and 7-years-old and families were required to speak English and be free from a developmental concern that could prevent the completion of study procedures. Research examining synchrony among caregiver-child dyads have noted differences in male and female caregiver emotional and neurophysiological responding (Kochanska et al., 2008; Liu et al., 2024; Lunkenheimer et al., 2021a, b). Therefore, to reduce variability attributed by caregiver gender, only female caregivers were recruited for the study. Approximately two months into active data collection, inclusion criteria were adjusted to only include additional participants that identified members of an underrepresented racial or ethnic group to increase racial and ethnic diversity within the sample. Regarding child race, mothers reported that 40% of children identified as White (Non-Hispanic), 27.5% Black/African American, 15.0% Other (predominantly reported Biracial), and 2.5% Asian. 15.0% of mothers reported that their child was of Hispanic, Latinx, or Spanish heritage. The majority of mothers endorsed being married (65.0%) with the remaining mothers reporting being single (28.7%), divorced (3.8%), or separated (2.5%). Regarding education level, mothers reported completing a bachelor’s degree (32.5%), graduate degree (37.5%), some college (23.80%), a high school diploma (3.8%), and 2.6% reported having less than a high school diploma. Approximately 11.3% of mothers reported a family income of less than $30,000, 31.3% of mothers reported earning between $30,000 and $59,999, 23.7% reported earning between $60,000 and $89,999, and 33.8% of mothers endorsed a familial income of greater than $90,000.

### Procedures

Study procedures were approved and in accordance with the University of Georgia’s Institutional Review Board (IRB). Participants were recruited from the local community in the southeastern United States through flyers, social media postings, in-person community events, and participant referrals. Interested families were screened over the phone for eligibility and if they met criteria, were scheduled for a two-hour assessment in the laboratory. Mothers and children provided written consent and assent, respectively, prior to completing study procedures. Mothers then completed a battery of questionnaires in relation to their affect experiences and child’s socioemotional functioning. Electrodes and fNIRS caps were then placed on mothers and their children (see below for data acquisition, cleaning, and processing procedures). Dyads were instructed to watch a 2-minute video of calming nature scenery while baseline estimates of resting heart rate and blood-oxygenation levels were collected continuously using BIOPAC MP160 System for physiological data and NIRx for neural activity. Dyads then engaged in a 5-minute mild stress task where children were asked to build a developmentally advanced LEGO figure. Mothers were informed that they could provide verbal assistance to the child but could not physically touch the LEGOs. Participants were told they had 5 min to complete the figure, and a visual timer was displayed with frequent audio cues to remind the dyad of the time remaining on the task. See Supplemental Fig. 1 for a picture of the task. Video recordings for behavioral observations, RSA, and fNIRS were collected continuously throughout the task. Timing was standardized across each assessment such that each dyad had the same time increments between tasks although visits occurred during different times of the day to accommodate participant availability. At the conclusion of the study, participants were informed that the child did not have all of the pieces needed to complete the LEGO figure. Families were compensated $100 in cash for their participation in the study an additional $20 for every family they referred who enrolled in study procedures.

#### RSA Data Acquisition

Mother and child physiological data was collected during both the baseline and mother-child stress task using BIOPAC’s *AcqKnowledge* software within the BIOPAC MP160 System. Three disposable electrocardiogram (ECG) electrodes were placed on each mother-child dyad in the following regions: the right collarbone (i.e., right clavicle area) and at the base of the rib region on the left and right sides of the body. Electrodes were then connected to the wireless BioNomadix ECG transmitter belts that monitored heart rate throughout study procedures. Once data collection was complete, trained research assistants used CardioPeak + Segmenter to divide the ECG recordings into small segments (i.e., 1 Hz). An artifact correction was then conducted using CardioEdit software, which enabled midbeat corrections by adding, splitting, or averaging beats as needed (Brain Body Center, University of Illinois at Chicago). After cleaning, interbeat intervals (IBIs) were extracted and a bandpass filter was applied to isolate the relevant frequency range. A continuous measure of RSA was computed as a natural logarithm of the variance within a 15-second sliding window (Abney et al., 2021).

#### FNIRs Data Acquisition

For Functional Near-Infrared Spectroscopy (fNIRS), we used the NIRSport 16–16 device (NIRx Medizintechnik GmbH, Germany) to collect data on oxyhemoglobin (HbO), deoxyhemoglobin (HbR), and total hemoglobin (HbT) in mother-child dyads. Each dyad was fitted with an electroencephalography (EEG) cap structured according to the 10–20 system. The probe sets consisted of 8 sources and 8 detectors, resulting in a total of 16 channels per individual and was consistent for all participants. The optodes were positioned over the left and right frontal regions. For source-detector channel arrangement, the devFOLD toolbox (Fu & Richards, 2021) was used to optimize Channel placement accuracy across child and adult montages. The device emitted infrared light at wavelengths of 760 nm and 850 nm and data was collected at a sampling rate of 7.81 Hz. For a pictural representation of the optode placement, see Supplemental Fig. 2.

### Measures

#### Maternal Affect Experiences

Mothers completed the 20-item Positive and Negative Affect Schedule (PANAS-SF, Watson et al., 1988) to measure the extent of positive and negative affect they felt over the past week. Mothers rated 10 specific positive emotions (e.g., “Proud,” and “Enthusiastic”) and 10 negative emotions (e.g., “Ashamed,” “Nervous,”) using a 5-point Likert scale (1 = *Very Slightly or Not at All* to 5 = *Extremely*). Reliability was satisfactory (*α* = 0.83 and 0.91 for PA and NA, respectively). All mothers successfully completed this questionnaire.

#### Child Emotion Dysregulation

Mothers completed the Negative Lability subscale of the Emotion Regulation Checklist (ERC, Shields & Cicchetti, 1997) to measure children’s frequency of negative lability (*α* = 0.80). The subscale consists of 15 items such as “Is easily frustrated,” “Is prone to angry outbursts/tantrums easily,” and “Is prone to disruptive outbursts of energy or exuberance” rated on a 4-point Likert scale (1 = *Never* to 5 = *Always*). An average score was calculated across the 15 items and used in analyses. No children were missing questionnaire data.

### RSA Data Cleaning and Processing

During processing, RSA was sampled at 1 Hz. However, to align with prior work recommending RSA be analyzed in 30-second epochs to best capture physiological reactivity to stress (Berntson et al., 1997; Fisher & Woodward, 2014; Xu et al., 2024a), RSA were aggregated and averaged every 29 samples. This resulted in 10 timepoints for each person as this was the closest approximate to 30-second epochs. Mother-child RSA synchrony was computed using Bayesian Structural Equation Modelling (Asparouhov & Muthén, 2019) following Xu et al. (2024b)’s procedure in *MPlus* 8.0. The model also used the average RSA of the last 29 timepoints in the baseline as the reference point and controlled for the auto-regression of RSA. Six dyads had missing data due to an inability to clean/derive reliable physiological values, resulting in a total of 74 dyads with usable RSA synchrony data.

### FNIRs Data Cleaning, and Processing

The data processing procedure for fNIRs was based on steps outlined by Nguyen et al. (2020). The dataset was evaluated for missing values. A visual inspection of all Channels was conducted to identify and exclude those with significant issues. Additionally, an automated Channel pruning process was employed to detect and remove poor-quality channels using criteria such as signal integrity. Once the data were cleaned, they were converted into optical density values to measure the absorption of light by brain tissue. Motion artifacts caused by participant movement were addressed through spline interpolation and wavelet-based correction methods. Then, filters were applied to eliminate irrelevant fluctuations. The cleaned data was then converted into concentrations of HbO, HbR, and HbT, and downsampled from 7.81 Hz to 1 Hz. Finally, the fNIRS data were temporally aligned with the baseline and LEGO tasks. Of the 80 dyads, 65 were useable for further analysis. Each participant of the 65 dyads used 8 channels (4 for the left and right dorsolateral prefrontal cortex) out of the total 18-channel montage (8 out of 18 channels used). Out of the 8 Channels used for each dyad, there were 1,040 total Channels analyzed across 65 dyads (65 dyads x 2 participants x 8 channels) which resulted in 81.25% useable data from the total possible dataset. The 15 (18.75%) subjects that were excluded were removed due to poor signal-to-noise ratio, excessive motion artifacts, or study interruptions.

For analysis, brain activity measures from the left and right dorsolateral prefrontal cortex were aggregated across fNIRS channels for both mothers and children. The resulting columns represent these aggregated activity measures. HbR is typically less susceptible to artifacts from extracerebral sources, provides greater spatial specificity, and is less influenced by autonomic nervous system activity (Kayhan et al., 2022; Kirilina et al., 2013; Piazza et al., 2020; Tachtsidis & Scholkmann, 2016). Conversely, the HbO signal is more sensitive to changes in regional cerebral blood flow and exhibits a higher signal-to-noise ratio (Hoshi, 2007; Kinder et al., 2022; Pan et al., 2017). Given signal-type has specific advantages, the present study reported both HbR and HbO results as recommended by the literature (Boas et al., 2014; Yücel et al., 2021; see Tables 2 and 4).

### FNIRS Neural Synchrony Analysis

Wavelet transform coherence (WTC) of fNIRS time-series data was used as an estimate of brain-to-brain synchrony for each mother-child dyad (Grinsted, et al., 2004). More specifically, WTC was used to compute the cross-correlation between each dyad’s aggregated fNIRS time series, as a function of time and frequency. This computation evaluates overall coherence patterns in the dyad’s brain activity, while considering both phase-lagged and in-phase correlations (Nguyen et al., 2021a, b). Higher coherence values indicate a higher correlation between mother and child brain activity across task duration and a chosen frequency range. However, it is worth noting that while WTC is useful in identifying similar patterns of change in brain activity, it is unable to detect directionality, such that coherence values alone may not be used to determine whether synchrony is being driven by in-phase, anti-phase, or lagged synchronization. WTC was calculated with the WTC function from the “biwavelet” package in R (v0.20.22; Gouhier et al., 2024), using the default Morlet wavelet. Coherence values were averaged across a frequency band of interest (FOI) of 0.02–0.1 Hz (corresponding to periods of 10–50 s) for the duration of the 5-minute stress task for each of the aggregated dlPFC signals, resulting in 4 coherence values for each dyad (right and left dlPFC HbO and HbR). This FOI was chosen due to its identification as most relevant to parent-child problem solving tasks and reduction of artifacts from physiological noise produced by respiration (~ 0.2–0.3 Hz), cardiac pulsation (~ 1 Hz), and Mayer waves (~ 0.1 Hz; Nguyen et al., 2021a, b).

### FNIRS Neural Synchrony Random Pairing Control Analysis

To test for the significance of neural synchrony analyses above spurious correlations, we conducted random pair analyses in the same region (right and left dlPFC) and signal type (HbR and HbO) with a permutation approach. To that end, each mother was paired with a random child from another dyad 100 times, and coherence was averaged for each region and signal type across all random pairings. The random pair coherence values were entered in the same analyses as the true dyad coherence values to validate that results were due to synchrony between true dyads and not random chance or signal noise. Values for true mother-child dyads are expected to be higher (i.e., more strongly related) when compared to values observed in random pairings.

### Data Analysis Plan

Correlation analyses were conducted to investigate relations between study variables using SPSS Version 29.0.2.0. Then, in ten separate models, the present study investigated the association between mothers’ positive and negative affect experiences and children’s negative lability as moderated by RSA, right, and left dlPFC synchrony. Two models assessed the moderating effect of RSA synchrony on maternal affect experiences with child negative lability, one for maternal positive and the other for maternal negative affect experiences. The next four models examined the moderating effect of right and left dlPFC synchrony independently using the HbR signal. The final four models again examined the moderating effect of right and left dlPFC synchrony using the HbO signals. Across all models, the interaction between maternal affect experiences and synchrony were examined in relation to child negative lability. Given the different scales used for the independent variable and the moderator, variables were mean-centered prior to creating the interaction term. Maternal negative affect experiences was entered as a covariate for models in which maternal positive affect experiences was the independent variable to control for spurious results, and vice versa. Child gender and income were also entered as covariates across all models. Interaction effects that crossed the threshold of significance (*p* <.050) were further examined using the Johnson-Neyman technique to explore the regions of significance.

## Results

Descriptive statistics and bivariate correlations between study variables can be found in Table 1. For a full list of regression analyses, refer to Tables 2 and 3.

**Table 1:** Descriptive statistics

Variable	M (SD)	1	2	3	4	5	6	7	8	9
1. Gender	-	-								
2. Income	-	− 0.09	-							
3. Maternal Positive Affect Experiences	36.21 (6.78)	0.27*	0.02	-						
4. Maternal Negative Affect Experiences	19.54 (7.26)	− 0.04	− 0.11	− 0.26*	-					
5. Child Emotion Dysregulation	1.79 (0.37)	− 0.07	− 0.02	− 0.18	0.43**	-				
6. RSA Synchrony	−0.01 (0.03)	0.11	− 0.06	0.18	− 0.14	− 0.23	-			
7. Right HbR dlPFC Synchrony	0.33 (0.05)	− 0.05	− 0.17	0.004	0.16	0.18	− 0.11	-		
8. Left HbR dlPFC Synchrony	0.33 (0.05)	0.11	− 0.02	0.18	0.000	− 0.002	0.18	0.08	-	
9. Right HbO dlPFC Synchrony	0.35 (0.05)	0.09	− 0.06	0.01	− 0.19	− 0.17	0.11	0.17	− 0.10	-
10. Left HbO dlPFC Synchrony	0.34 (0.06)	0.15	− 0.12	0.04	− 0.06	− 0.004	0.16	0.08	0.003	0.36**

Note. *RSA* Respiratory Sinus Arrythmia, *HbR* deoxyhemoglobin, *HbO* oxyhemoglobin, *dlPFC* Dorsolateral Prefrontal Cortex. ^*^p <.05, ^**^p <.01

**Table 2:** Moderation for maternal positive affect experiences models

Model	B	SE	β	*p*
*Moderator*: RSA Syncrhony	*R*^2^ = 0.28, *F*(6, 64) = 4.18, *p* =.001			
Maternal Positive Affect	−0.01	0.01	− 0.12	0.140
RSA Synchrony	13.92	7.06	− 0.24	0.053
Mat Pos Aff X RSA Sync	−0.47*	0.20	− 0.24	0.021
*Cov*: Maternal Negative Affect	0.02**	0.01	0.46	< 0.001
*Cov*: Child Gender	0.02	0.09	0.05	0.851
*Cov*: Income	0.01	0.01	0.09	0.461
*Moderator*: Right dlPFC Syncrhony (HbR Signal)	*R*^2^ = 0.29, *F*(6, 55) = 3.79, *p* =.003			
Maternal Positive Affect	0.08	0.04	0.06	0.059
Right dlPFC Syncrhony	9.40*	4.16	0.04	0.028
Mat Pos Aff X Right dlPFC Sync	−0.25*	0.12	− 0.24	0.041
*Cov*: Maternal Negative Affect	0.02**	0.01	0.38	0.003
*Cov*: Child Gender	0.04	0.09	0.10	0.680
*Cov*: Income	−0.01	0.02	− 0.02	0.892
*Moderator*: Left dlPFC Syncrhony (HbR Signal)	*R*^2^ = 0.31, *F*(6, 55) = 4.06, *p* =.002			
Maternal Positive Affect	0.13*	0.05	0.01	0.015
Left dlPFC Syncrhony	14.70*	5.62	0.10	0.012
Mat Pos Aff X Left dlPFC Sync	−0.39*	0.15	− 0.39	0.011
*Cov*: Maternal Negative Affect	0.02**	0.01	0.44	0.001
*Cov*: Child Gender	−0.10	0.09	− 0.26	0.229
*Cov*: Income	0.01	0.02	0.01	0.924
*Moderator*: Right dlPFC Synchrony (HbO Signal)	*R*^2^ = 0.24, *F*(6, 55) = 2.86, *p* =.017			
Maternal Positive Affect	0.04	0.05	.14	.477
Right dlPFC Synchrony	4.33	6.27	.07	.493
Maternal Positive Affect X Right dlPFC Synchrony	−0.14	0.16	-.12	.414
*Cov*: Maternal Negative Affect	0.02**	0.01	.38	.004
*Cov:* Child Gender	−0.02	0.09	-.06	.814
*Cov: *Income	−0.01	0.02	-.09	.479
*Moderator*: Left dlPFC Synchrony (HbO Signal)	*R*^2^ = 0.23, *F*(6, 55) = 2.75, *p* =.021			
Maternal Positive Affect	0.02	0.03	.08	.476
Left dlPFC Synchrony	3.25	3.61	.02	.371
Maternal Positive Affect X Right dlPFC Synchrony	−0.09	0.10	-.10	.379
*Cov:* Maternal Negative Affect	0.02**	0.01	.42	.002
*Cov:* Child Gender	−0.01	0.10	-.02	.928
*Cov*: Income	−0.01	0.02	-.06	.631

Note. *RSA* Respiratory Sinus Arrythmia, *Pos Aff* positive affect, *Sync* synchrony, *Cov* covariate, *dlPFC* Dorsolateral Prefrontal Cortex. ^*^p <.05, ^**^p <.01

**Table 3:** Moderation for maternal negative affect experiences models

Model	B	SE	β	*p*
*Moderator*: RSA Syncrhony	*R*^2^ = 0.26, *F*(6, 64) = 3.77, *p* =.003			
Maternal Negative Affect	0.03**	0.01	0.44	< 0.001
RSA Synchrony	−11.83*	5.17	− 0.25	0.026
Mat Neg Aff X RSA Sync	0.43	0.23	0.23	0.059
*Cov*: Maternal Positive Affect	−0.01	0.01	− 0.09	0.488
*Cov*: Child Gender	−0.01	0.09	− 0.03	0.901
*Cov*: Income	0.01	0.01	0.04	0.726
*Moderator*: Right dlPFC Syncrhony (HbR Signal)	*R*^2^ = 0.25, *F*(6, 55) = 3.00, *p* =.013			
Maternal Negative Affect	−0.03	0.05	− 0.37	0.521
Right dlPFC Syncrhony	−2.07	2.98	− 0.16	0.491
Mat Neg Aff X Right dlPFC Sync	0.16	0.16	0.17	0.312
*Cov*: Maternal Negative Affect	−0.01	0.02	− 0.10	0.856
*Cov*: Child Gender	−0.02	0.09	− 0.05	0.838
*Cov*: Income	−0.002	0.02	− 0.02	0.856
*Moderator*: Left dlPFC Syncrhony (HbR Signal)	*R*^2^ = 0.23, *F*(6, 55) = 2.75, *p* =.021			
Maternal Negative Affect	−0.02	0.04	− 0.41	0.703
Left dlPFC Syncrhony	−2.08	2.52	− 0.02	0.703
Mat Neg Aff X Left dlPFC Sync	0.11	0.13	0.12	0.376
*Cov*: Maternal Positive Affect	−0.01	0.07	− 0.09	0.507
*Cov*: Child Gender	−0.04	0.10	− 0.12	0.639
*Cov*: Income	−0.01	0.02	− 0.06	0.643
*Moderator*: Right dlPFC Sync (HbO Signal)	*R*^2^ = 0.26, *F*(6, 55) = 3.27, *p* =.008			
Maternal Negative Affect	0.08*	0.04	.31	.043
Right dlPFC Synchrony	3.07	2.57	.10	.237
Mat Neg Aff X Right dlPFC Sync (*int*)	−0.20	0.12	-.18	.113
*Cov:* Maternal Positive Affect	−0.01	0.01	-.12	.381
*Cov:* Child Gender	−0.03	0.09	-.08	.726
*Cov*: Income	−0.01	0.02	-.04	.700
*Moderator*: Left dlPFC Synchrony (HbO Signal)	*R*^2^ = 0.23, *F*(6, 55) = 2.68, *p* =.024			
Maternal Negative Affect	−0.002	0.03	-.45	.964
Left dlPFC Synchrony	−1.43	2.38	-.01	.551
Mat Neg Aff X Right dlPFC Sync	0.07	0.11	.09	.499
*Cov*: Maternal Positive Affect	−0.004	0.01	-.08	.546
*Cov*: Child Gender	−0.03	0.10	-.06	.788
*Cov*: Income	−0.01	0.02	-.08	.554

Note. *RSA* Respiratory Sinus Arrythmia, *Neg Aff* negative affect, *Sync* synchrony, *Cov* covariate, *dlPFC* Dorsolateral Prefrontal Cortex. ^*^p <.05, ^**^p <.01

### Maternal Positive Affect Experiences

#### RSA Synchrony Moderation

**Fig. 1:**
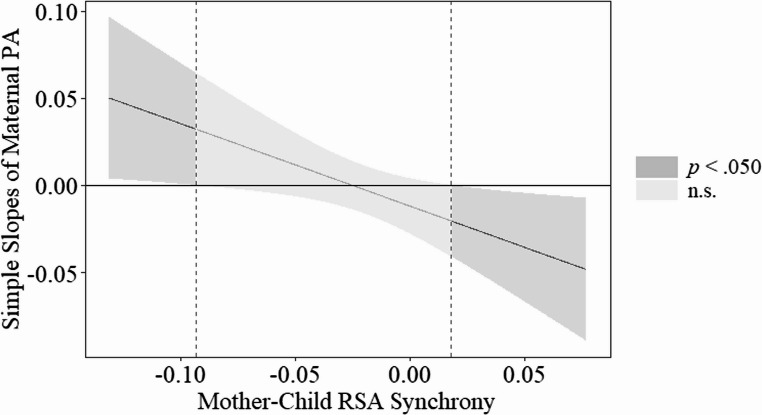
J-N plot for maternal positive affect and RSA synchrony

The overall model was statistically significant (*R*^2^ = 0.28, *F*(6, 64) = 4.18, *p* =.001). In line with study hypotheses, the interaction between maternal positive affect experiences and RSA synchrony in relation to child emotion dysregulation was significant (*B* = −0.47, *SE* = 0.20, β = − 0.24, *p* =.021). Given the significant interaction term, the J-N technique was used to identify the regions of significance. Results suggested the relation between maternal positive affect experience and child emotion dysregulation was negative at and above values of 0.018 of RSA synchrony, and it was positive at and below values of −0.09 of RSA synchrony. See Fig. 1 for graphical depiction.

#### Right dlPFC Synchrony Moderation (HbR Signal)

**Fig. 2:**
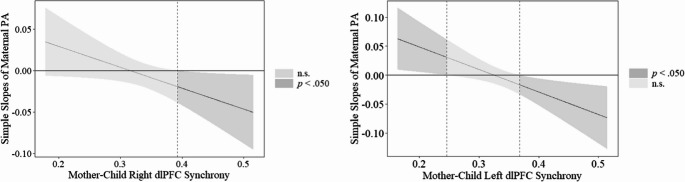
J-N plots for maternal positive affect and left and right dlPFC synchrony

The overall model was statistically significant (*R*^2^ = 0.29, *F*(6, 55) = 3.79, *p* =.003). The interaction between maternal positive affect experiences and right dlPFC synchrony was significant (*B* = −0.25, *SE* = 0.12, β = − 0.24, *p* =.041). Using the J-N technique, a stronger negative relation between maternal positive affect experiences and child emotion dysregulation was found when right dlPFC synchrony was more positive (raw scores ≥ 0.39, *p* =.050). Conversely, the relation between maternal positive affect experiences and child emotion dysregulation was nonsignificant at weaker and negative levels of right dlPFC synchrony (i.e., raw scores ≤ 0.38, *p* =.064; Fig. 2).

#### Left dlPFC Synchrony Moderation (HbR Signal)

The overall model was statistically significant (*R*^2^ = 0.31, *F*(6, 55) = 4.06, *p* =.002). Similar to the findings for right dlPFC synchrony, the interaction between maternal positive affect experiences and left dlPFC synchrony was significant (*B* = −0.39, *SE* = 0.15, β = − 0.39, *p* =.012). With the J-N technique, the negative relation between maternal positive affect and child emotion dysregulation was strongest in the context of strong, positive left dlPFC synchrony (raw score ≥ 0.37, *p* =.050). Although the association between maternal positive affect experiences and child emotion dysregulation was weakest at low, positive levels of left dlPFC synchrony, this association occurred prior to left dlPFC synchrony becoming negative (raw score ≤ 0.25, *p* =.050). Refer to Fig. 2 for right and left dlPFC effects with the HbR signal.

#### Right dlPFC Synchrony Moderation (HbO Signal)

Due to the discrepant literature regarding whether to report HbR versus HbO signal results when conducting fNIRs analyses, the current study reported both for the sake of clarity. Although the overall model was significant (*R*^2^ = 0.24, *F*(6, 55) = 2.86, *p* =.017), the interaction between maternal positive affect experiences and right dlPFC synchrony with the HbO signal was nonsignificant (*B* = −0.14, *SE* = 0.16, β = − 0.18, *p* =.414).

#### Left dlPFC Synchrony Moderation (HbO Signal)

The overall model was significant (*R*^2^ = 0.23, *F*(6, 55) = 2.75, *p* =.021) but the interaction between maternal positive affect experiences and left dlPFC synchrony with the HbO signal was nonsignificant (*B* = −0.09, *SE* = 0.10, β = − 0.10, *p* =.379).

### Maternal Negative Affect Experiences

#### RSA Synchrony Moderation

The overall model was statistically significant (*R*^2^ = 0.26, *F*(6, 64) = 3.77, *p* =.003). Contrary to hypotheses, the interaction between maternal negative affect experiences and RSA synchrony was nonsignificant (*B* = 0.43, *SE* = 0.23, β = − 0.24, *p* =.059). However, given the near significance, the interaction was probed using the J-N technique. The positive association between maternal negative affect experiences and child emotion dysregulation grew stronger as RSA synchrony became more positive (raw score ≥ −0.03, *p* =.050).

#### Right dlPFC Synchrony Moderation (HbR Signal)

Although the overall model was statistically significant (*R*^2^ = 0.25, *F*(6, 55) = 3.00, *p* =.013), the interaction between maternal negative affect experiences and right dlPFC synchrony was nonsignificant (*B* = 0.16, *SE* = 0.16, β = 0.17, *p* =.312).

#### Left dlPFC Synchrony Moderation (HbR Signal)

The overall model was statistically significant (*R*^2^ = 0.23, *F*(6, 55) = 2.75, *p* =.021). However, the interaction between maternal negative affect experiences and left dlPFC synchrony was nonsignificant (*B* = 0.11, *SE* = 0.13, β = 0.12, *p* =.376).

#### Right dlPFC Synchrony Moderation (HbO Signal)

Although the overall model was significant (*R*^2^ = 0.26, *F*(6, 55) = 3.27, *p* =.008), the interaction between maternal negative affect experiences and right dlPFC synchrony with the HbO signal was nonsignificant (*B* = −0.20, *SE* = 0.12, β = − 0.18, *p* =.113).

#### Left dlPFC Synchrony Moderation (HbO Signal)

The overall model was significant (*R*^2^ = 0.23, *F*(6, 55) = 2.68, *p* =.024) but the interaction between maternal negative affect experiences and left dlPFC synchrony with the HbO signal was nonsignificant (*B* = 0.07, *SE* = 0.11, β = 0.09, *p* =.499).

### Random Pairing Check for dlPFC Synchrony Moderation (HbR Signal)

As a test of the interaction effects between maternal positive affect experiences and dlPFC synchrony in the right and left hemispheres, all moderations were computed a second time with random pairings to ensure significant effects for both HbR and HbO signals were due to dlPFC synchrony between mothers and their children, rather than a product of noise and/or randomness. The interaction between maternal positive affect experiences and dlPFC synchrony with random pairings using the HbR signal was nonsignificant in the right (*B* = −0.40, *SE* = 0.50, β = −0.24, *p* =.437) and left (*B* = −0.73, *SE* = 0.39, β = −0.24, *p* =.0.067) hemispheres. The same nonsignificant pattern emerged for the interaction between maternal negative affect experiences with right (*B* = −0.88, *SE* = 0.44, β = −0.24, *p* =.053) and left (*B* = −0.68, *SE* = 0.41, β = −0.24, *p* =.106) dlPFC synchrony using the HbR signal.

### Random Pairing Check for dlPFC Synchrony Moderation (HbO Signal)

The interaction between maternal positive affect experiences and dlPFC synchrony when measured using the HbO signal with random pairings was nonsignificant in the right (*B* = 0.12, *SE* = 0.60, β = 0.03, *p* =.839) and left (*B* = 0.99, *SE* = 0.54, β = 0.23, *p* =.070) hemispheres. The same nonsignificant pattern emerged for the interaction between maternal negative affect experiences with right (*B* = 0.41, *SE* = 0.46, β = 0.11, *p* =.376) and left (*B* = −0.60, *SE* = 0.54, β = −0.14, *p* =.274) dlPFC synchrony using the HbO signal. Results from random pairings bolster confidence that the significant interactions noted above with dlPFC synchrony are products of mother-child neural synchrony and not chance.

## Discussion

The present study builds substantively on extant literature by using a multimethodological, multilevel approach to examine the association of maternal affect experiences as well as mother-child physiological and neural processes on children’s emotion dysregulation - a transdiagnostic indicator of psychopathology (Beauchaine & Hinshaw, 2020; Fonagy et al., 2017). The early school-age period reflects a critical developmental stage by which to examine these processes given children during this stage of development still benefit from external regulation while also shifting towards greater internal regulatory processes (Senehi & Brophy-Herb, 2020). Mothers’ own affect experiences play a significant role in how they interact and express emotions with their children (Deater-Deckard et al., 2016; Feng et al., 2008; Halberstadt et al., 1993). Mother-child interactions during the school-age period then provide opportunities for child emotional development (Bell, 2020; Eisenberg et al., 1998). Emotion dysregulation, or difficulty in the experience, expression, and/or modulation of emotion, underlies a host of mental health conditions across youth development (Beauchaine & Hinshaw, 2020; Fonagy et al., 2017; McLaughlin & Gabard-Durnam, 2022). Given emotion dysregulation is a transdiagnostic indicator of psychopathology, the present study examined the relation between maternal affect experiences and child emotion dysregulation, as moderated by mother-child neurophysiological synchrony.

Consistent with extant literature, positive maternal affect experiences were generally associated with low child emotion dysregulation, whereas negative maternal affect experiences were linked to high child emotion dysregulation (Tan et al., 2019; Zimmer-Gembeck et al., 2022). For children in the early school-age period, warm, supportive emotional environments are essential to children learning how to appropriately express and regulate their own emotions across interaction contexts (Eisenberg, 2020; Ratliff et al., 2022). Mothers who report a preponderance of positive affect likely create a supportive and secure parent-child interaction environment, which facilitates child emotional expression and fosters healthy emotion regulation development (Morris et al., 2018). Conversely, maternal negative affect experiences have been linked to high emotional reactivity and unsupportive parenting behaviors, which may compromise children’s ability to safely express emotions and disrupt child emotion regulation (Edler et al., 2024).

### Maternal Positive Affect Experiences

In support of hypotheses, there was also a negative association in the context of positive RSA synchrony. This negative association was also found for right and left dlPFC synchrony. Positive RSA synchrony has been positively associated with both mother and child individual and shared positive affect expression in a sample of 8-to-10-year-old children and their mothers (Zehra Capraz et al., 2023). Similarly, Xu and colleagues (2024b) found that the link between parent-child RSA synchrony and child emotion regulation was positive in the context of high parental emotional support (e.g., likely also indicative of maternal positive affect experiences) in a sample of Chinese school-age children. In short, prior work has shown the benefits of mother positive affect experiences and RSA synchrony for maximizing positive child functioning. Interestingly, and contrary to hypotheses, there was also a positive relation between maternal positive affect experiences and child emotion dysregulation at low values of RSA synchrony. Regarding this perplexing association, perhaps at low levels of positive RSA synchrony, children do not benefit from the positive affect experiences of mothers or were unable to experience these positive effects, resulting in increased emotion dysregulation. Findings from the current study expand on this work by showing the benefits of mother positive affect for minimizing child emotion dysregulation, an effect that is strengthened in the context of high levels of positive RSA synchrony. It may be especially beneficial for children of well-regulated mothers to synchronize physiologically with their caregiver. Physiological synchrony at high levels in the context of maternal modeling of positive emotion may buffer against the development of child emotion dysregulation as these processes scaffold healthy self-regulation. Longitudinal designs can examine the potential long-term protective benefits of an emotionally-positive caregiving environment in combination with physiological synchrony for child psychopathology symptoms (McLaughlin & Gabard-Durnam, 2022).

Findings provide direct empirical support for the extension of the parent-child emotion regulation dynamics model (Ratliff et al., 2022), suggesting that the relation between maternal affect experiences and child emotion dysregulation specifically varies depending on the neural synchronization of mothers with their children. With respect to positive dlPFC synchrony, in line with earlier research, there was a positive link between positive dlPFC synchrony with HbR signals and child emotion dysregulation (Miller et al., 2019; Nguyen et al., 2020). While preliminary work has established the value in dlPFC synchrony across cooperative, competitive, and individual tasks for adaptive child socioemotional functioning (Miller et al., 2019; Nguyen et al., 2020; Reindl et al., 2018), we extend this literature by demonstrating the value of positive dlPFC synchrony during a mildly stressful and emotionally laden parent-child interaction task. Mirroring the results for physiological synchrony, current findings suggest that when children are neurally-attuned to their mothers during a mildly stressful task, their symptoms of emotion dysregulation are significantly reduced. DlPFC activation represents neural underpinnings in relation to one’s self-regulation (Lee & Williams, 2024; Monsell, 2003). Thus, when a mother-child dyad is able to synchronously move with one another during a stressful time, this has implications for individual child self-regulation. Although extent literature has linked maternal adaptive self-regulation to positive child adjustment (Are & Shaffer, 2016; Morris et al., 2018; Ratliff et al., 2022; Tan et al., 2019), present findings suggest that dlPFC synchrony and maternal positive affect experiences are synergistically related to a robust transdiagnostic indicator of child psychopathology.

In regards to the lateralization of the dlPFC, prior literature has linked individual left dlPFC activation to experiences of positive and negative mood whereas right dlPFC has been linked to broader emotional processing (Maeoka et al., 2012; Pena-Gomez et al., 2011; Sanchez-Lopez et al., 2018). Although lateralization of dlPFC activation has been important in understanding individual processes, the current findings regarding the link between maternal positive affect experiences and child emotion dysregulation were consistent for left and right dlPFC synchrony. However, the interaction between maternal positive affect experiences and left dlPFC synchrony was stronger when compared to that of right dlPFC synchrony. Individual-level left dlPFC activation has been related to efficient emotion regulation and affect experiences, which is consistent with the extended parent-child emotion regulation dynamics model and is echoed in the current findings (De Raedt et al., 2015; Ratliff et al., 2022). Prior work suggests that mothers’ intrapersonal affect experiences shape how they interact with their children and, in turn, influence children’s abilities to regulate their own emotions (Deater-Deckard et al., 2016; Feng et al., 2008; Halberstadt et al., 1993). Results build upon prior literature which suggests that maternal affect experiences directly relate to children’s emotion dysregulation and that this association varies depending on dyadic indicators. While this relation has been theorized to vary by neurophysiological markers, the present study provides empirical support for this proposed association.

HbO synchrony did not show a comparable pattern of results as HbR synchrony. This discrepancy may be attributed to differential signal sensitivity. HbR has a lower amplitude than HbO. Nonetheless, HbR has been shown to be less sensitive to extracerebral hemodynamics and systemic physiological noise (e.g., scalp blood flow, respiration), potentially making it a more specific marker of localized cortical activity in naturalistic interaction contexts (Tachtsidis & Scholkmann, 2016; Kirilina et al., 2013). In contrast, HbO typically exhibits a higher signal-to-noise ratio but is also more susceptible to these confounds, which can obscure task-related neural signals. That said, the absence of HbO findings should be interpreted with caution, and future work is needed to determine under what conditions HbO and HbR may diverge in parent-child neural synchrony research.

### Maternal Negative Affect Experiences

Contrary to hypotheses, the interactions between maternal negative affect experiences and RSA synchrony as well as dlPFC synchrony (both left and right) were nonsignificant. However, the interaction between maternal negative affect experiences and RSA synchrony neared significance (*p* =.059), so the moderation was probed exploratorily. As expected, as RSA synchrony became more positive (raw score ≥ −0.03), the relation between maternal negative affect experiences and child emotion dysregulation was strengthened, suggesting that children show increasing emotion dysregulation as mothers’ self-reported negative affect experiences increase. During the early school-age period, children are highly attuned to the affect experiences and regulatory patterns of their parents and these observations can influence their own emotional development (Davis et al., 2015). For children with mothers reporting high degrees of negative affect experiences, children are likely observing more frequent emotional dysregulation when compared to children with mothers reporting lower levels. Domains of self-regulation (e.g., behavioral, physiological, emotional) do not operate in isolation but rather, are theorized to work as a coordinated system (Blair & Ku, 2022). Thus, if a mother is experiencing high degrees of negative affect, and the child becomes synchronized physiologically with her, this concordance likely exacerbates the child’s emotion dysregulation. Albeit only marginally significant, current findings provide empirical support for the notion that maternal negative affect experiences coupled with positive RSA synchrony confers increased risk for child maladjustment.

Contrary to hypotheses, there was no significant interaction between maternal negative affect experiences and neural synchrony in relation to child emotion dysregulation. This finding is perplexing particularly in light of other significant associations noted above. Perhaps the lack of effects could be due to the lower reported rates of maternal negative affect experiences (*M* = 19.54) when compared to positive affect (*M* = 36.21) in combination with a lower sample size for dlPFC synchrony. Decreased variance coupled with fewer data points could result in decreased power to detect statistically significant effects (Preacher et al., 2007). When looking at Pearson’s *r* values in Table 1, although not significant, all indicators of neural synchrony were negatively related to maternal positive affect experiences. The exact opposite appears to have emerged regarding the relation between maternal negative affect experiences and neural synchrony indices. This pattern of effects may suggest that a significant interaction may emerge with a larger sample size, though additional studies are needed to test that claim.

## Limitations

The current study benefitted from capturing the frequency of mother-reports of positive and negative affect experiences over the past week, and included the opposing maternal affect as a covariate in analyses. However, the PANAS measure does not capture the intensity level of trait positive and negative affect experiences or the degree of affect experienced specifically when interacting with one’s child. Thus, the measure is unable to determine whether high maternal emotional reactivity generally, or elevated affect specific to the parent-child context, might help explain the current findings. Finally, the mildly-stressful mother-child interaction task focused on the construction of a developmentally-advanced figure. A task designed to specifically elicit joint affect experiences (i.e., discussion of a sad topic) may provide further understanding and context for the links between maternal affect experiences and child emotion dysregulation.

## Conclusions

The current study adopted a multimethodological, multilevel approach to understanding the interaction of individual maternal affect experiences with dyadic parent-child neurophysiological indicators in relation to child emotion dysregulation, a transdiagnostic indicator of psychopathology (Beauchaine & Hinshaw, 2020). Results suggest that at high levels of positive RSA synchrony and right and left dlPFC synchrony, maternal positive affect experiences were associated with decreased child emotion dysregulation. Conversely, at low levels of positive RSA synchrony and right and left dlPFC synchrony, maternal positive affect experiences related to increased child emotion dysregulation. Results suggest that children who synchronize neurophysiologically with mothers reporting positive affective experiences may be less likely to exhibit emotion dysregulation. The results are relatively novel and the literature on neurophysiological synchrony is still developing. Nonetheless, findings suggest potential clinical implications with more research. Evidence-based treatments focused on enhancing positive parent-child interactions may have spillover effects on behavioral and neurophysiological processes that benefit parent-child dynamics and promote adaptive child emotion regulation (Im-Bolter et al., 2015). Further, results provide support for investigating dyadic approaches to biofeedback or neurofeedback therapy (Darling et al., 2020). These treatments may be particularly relevant within the early-school age period, when children experience increasing challenges to regulate emotions but still rely on caregivers for support. Through adopting a neurophysiological approach to understanding synchrony, the present study empirically demonstrated that multilevel synchrony processes have important implications for understanding a potent transdiagnostic indicator of child psychopathology.

## Supplementary Information

Below is the link to the electronic supplementary material.

Supplementary File 1

## Data Availability

Data are available upon reasonable request from the corresponding author.
