# Supplementary material for: Mother-Child Neurophysiological Synchrony Moderates the Relation Between Maternal Affect Experiences and Child Emotion Dysregulation
Source: Res Child Adolesc Psychopathol. 2025 Oct 20;53(12):1987–2002. doi: 10.1007/s10802-025-01380-4 (PMC12718249; doi:10.1007/s10802-025-01380-4)
Supplement: Supplementary file 1 [file 10802_2025_1380_MOESM1_ESM.docx]

**Supplemental Appendices**

**Supplemental Figure 1**

*Pictural Representation of Mother-Child Dyadic LEGO Task*


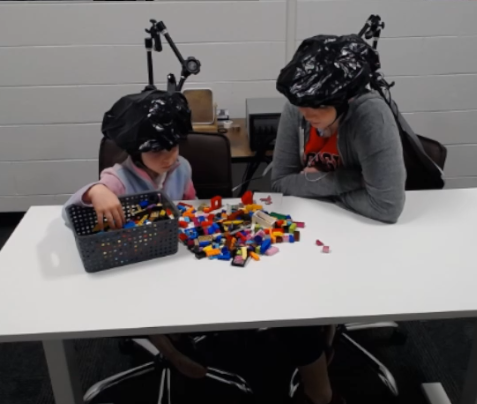


**Supplemental Figure 2**


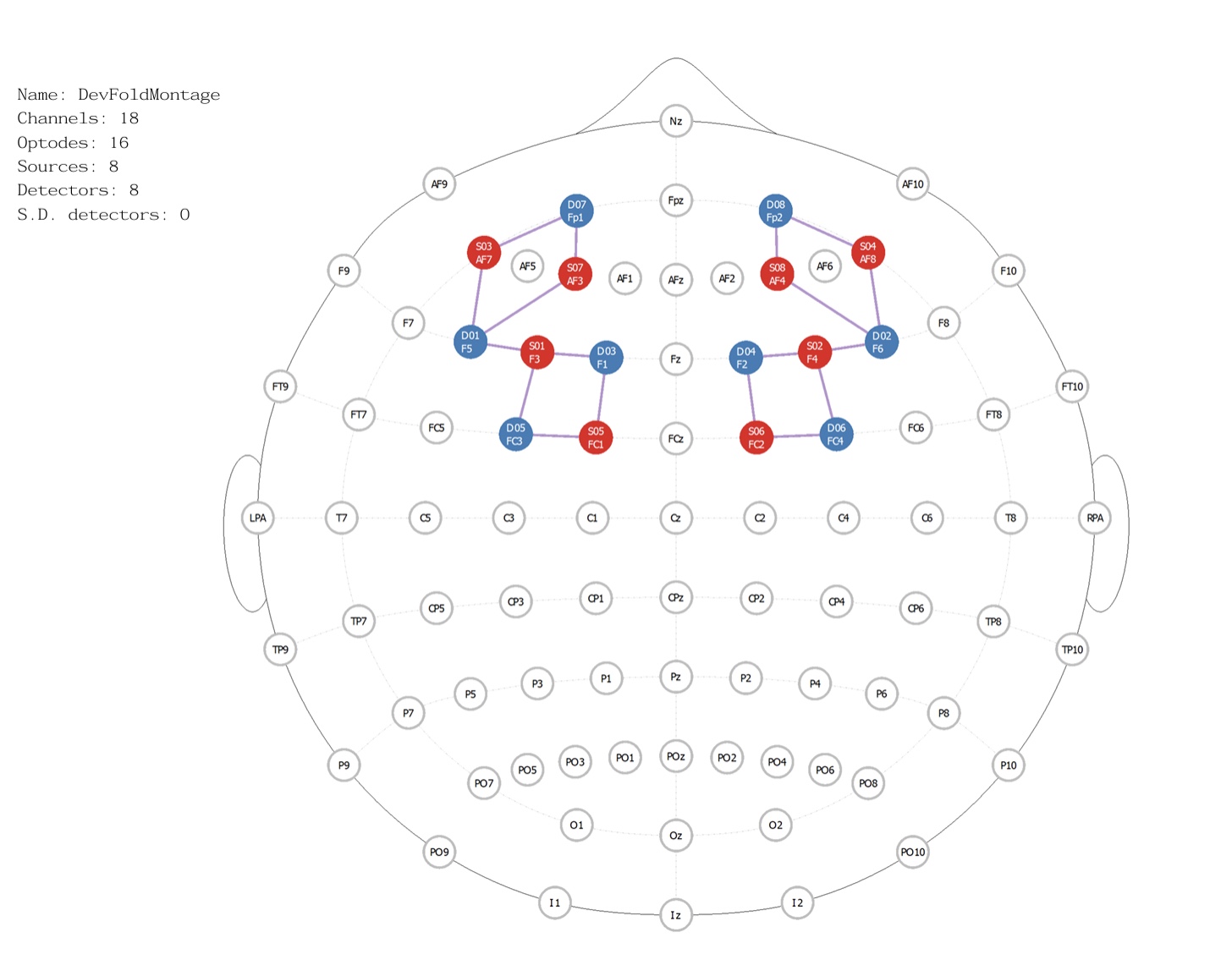
*fNIRs Optode Placement Visual*
